# Enhancing community engagement in Patient-Centered Outcomes Research: Equipping learners to thrive in translational efforts

**DOI:** 10.1017/cts.2021.835

**Published:** 2021-08-13

**Authors:** Jennifer M. Poger, Andrea E. Murray, Emily A. Schoettler, Evelyn S. Marin, Betsy B. Aumiller, Jennifer L. Kraschnewski

**Affiliations:** 1 Department of Medicine, Penn State College of Medicine, Hershey, PA 17033, USA; 2 Department of Public Health Sciences, Penn State College of Medicine, Hershey, PA 17033, USA

**Keywords:** Community engagement, stakeholder education, translational research, patient-centered outcomes research, community health workers

## Abstract

Community engagement is a critical component of translational research. Innovative educational approaches to support meaningful involvement of stakeholders in clinical research allows for bidirectional learning and greater engagement in translational efforts. Our Penn State Community-Engaged Research Core (CeRC) team has developed an innovative research curriculum for a variety of stakeholders, including patient partners, organizational representatives, and Community Health Workers (CHWs). This brief report will outline unique curricular approaches, guided by adult learning principles, to enhance stakeholder education and engagement in activities. Initial evidence of impact on learning is also reported.

## Rationale for Novel Application of Curricular Approach

Engaging nontraditional partners (i.e., patients, clinicians, policy-makers, organizational leaders, Community Health Workers [CHWs]) throughout the research process can further research priorities, enhance methodology, and accelerate translation to inform policy and practice and reduce disparities [1–3]. This essential spoke of the translational research wheel strengthens research activities through diverse stakeholder expertise and lived experience. A key goal of the Community-Engaged Research Core (CeRC) within the Penn State Clinical and Translational Science Institute is to broaden connections within the community and serve as a critical linkage for researchers and stakeholders [4]. Since stakeholders come from various educational and professional backgrounds, equipping them with tools and resources to support their roles as research partners is critical to successful engagement. Innovative educational approaches to support meaningful involvement of stakeholders in Clinical and Translational Science (CTS) allows for bidirectional learning and greater engagement in translational efforts. With the adult learner in mind, trainings and resources can be tailored to meet stakeholders where they are, providing relevancy and immediacy of knowledge in brief, interactive segments [5–7].

## Unmet Need or Educational Gap

Growing evidence suggests a positive resultant impact of stakeholder engagement in research [1–3,8,9]; however, there are limited readily available resources on how to effectively educate stakeholders to promote successful engagement in Patient-Centered Outcomes Research (PCOR) [10]. Community Health Workers (CHWs), for example, represent an untapped workforce well situated to bridge the community with various health services. The American Public Health Association defines a CHW as a “frontline public health worker who is a trusted member of, and/or has an unusually close understanding of, the community served” [11]. Community Health Workers have a proven track record of improving health outcomes, particularly for underserved populations, in a variety of community-based interventions [12,13]. Because of their intimate connections with the community, CHWs hold the potential to serve as liaisons between investigators and target populations in PCOR. Further, CHWs can engage hard-to-reach individuals within vulnerable communities, understand the context in which health problems exist, and serve as advocates for their communities’ needs in research [14]. Unfortunately, there remain several barriers preventing CHWs from engaging in PCOR, including: (1) lack of PCOR training in CHW certification programs, (2) lack of systems to connect CHWs with investigators interested in community-engaged research, and (3) recently, challenges associated with CHWs shifting to virtual work due to the COVID-19 pandemic [15].

The second gap is the utilization of stakeholders in effective research dissemination. Stakeholders representing community organizations, practice, or policy are well positioned to provide insight into research activities and can serve as conduits for results dissemination through their broad networks [16,17]. Additionally, patients and caregivers sit at the core of patient-centered outcomes and can transform research findings into relevant lay public resources, drawing from their intimate understanding of the day-to-day challenges of their condition [16]. Delivering a robust educational experience for partners new to PCOR can cast a wider dissemination net and facilitate increased uptake of study findings.

## Target Audience

Diverse stakeholders from multiple funded projects participated in our stakeholder educational programming to advance both their knowledge of PCOR and research engagement through project-tailored curriculum (Table 1). To illustrate, we outline two separate project examples highlighting our approach to stakeholder education. In the Patient-Centered Outcomes Research Institute (PCORI)-funded research project, Addressing Health Disparities with CHWs in COVID-19 (AHeaD with CHWs in COVID-19), CHWs (and other appropriate job categories, i.e., patient navigator, social worker) representing health systems, social service organizations, academic institutions, and businesses across the state of Pennsylvania were offered an innovative PCOR and remote work training, embedded in a long-standing CHW training program and as continuing education to previously trained CHWs. Both (1) a diverse Stakeholder Advisory Board (SAB) (n = 17) comprised of CHWs, CHW instructors, CHW managers, directors of health systems and organizations that utilize CHWs, and community-engaged researchers; and (2) a CHW Advisory Council (n = 11) comprised of expert CHWs closely connected with communities disproportionately affected by health disparities assisted in recruitment, collaboratively planned CHW trainings, and assisted in identifying a system to connect CHWs with researchers.

**Table 1.** Stakeholder educational programming characteristics

PCORI-funded research project	Stakeholders engaged	Educational program offered	Adult learning principles^a^	Examples from stakeholder education
AHeaD with CHWs in COVID-19	Community Health Workers (CHWs)	• Motivational interviewing during COVID-19 • Myth-busting • Creative community engagement strategies • Patient-Centered Outcomes Research (PCOR) concepts and research process topics	• Adults apply their diverse experiences to the learning environment • Adults are motivated to learn in problem-based environments	• Several attendees were active CHWs in their community and brought diverse experiences to the learning environment. Facilitators drew from these examples to enrich the learning process
PaTH to health: Diabetes study	Patients, clinicians, policy-makers, leaders from community agencies and regional/national professional organizations	• Research foundations and importance of community engagement • Community-Partnered Research Ethics Training (CPRET)^b^ • Just-In-Time (JIT) webinars on key research phases: data extraction, data analysis, results dissemination	• Adults need to know how and why content is relevant • Adults need to be active contributors to the learning process	• Modules provided examples of how content fit into the research process. For example, disseminating results is an important phase of research because it informs future work and can help translate findings more rapidly • There were several time points for questions and connections back to the specific research study. For example, during the research ethics training, partners discussed scenarios specific to natural experiments, including data quality and data security considerations

a **Bryan RL, Kreuter MW, Brownson RC.** Integrating adult learning principles into training for public health practice. *Health Promot Pract* 2009; **10**(4): 557–563.

b **Yonas MA, Jaime MC, Barone J, et al.** Community partnered research ethics training in practice: a collaborative approach to certification. *J Empir Res Hum Res Ethics* 2016; **11**(2): 97–105.

In the second PCORI-funded project, PaTH to Health: Diabetes, patient partners, and other community stakeholders representing policy, practice, and professional organizations were engaged in educational trainings at key intervals throughout the 5-year project. Project stakeholders (n = 22) were recruited from prior participation on advisory boards, clinician referrals, or were strategically invited to participate, given their shared focus of improving diabetes outcomes.

## Description of the Educational Method Utilized or Curricular Program

**Fig. 1.**
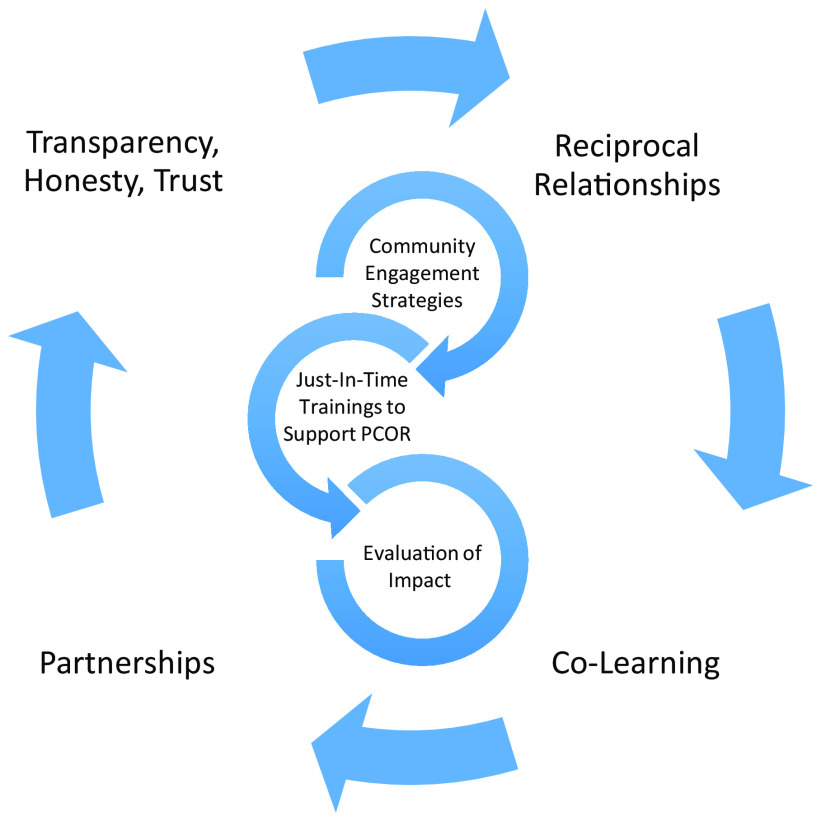
Patient-Centered Outcomes Research (PCOR) educational framework. *Note*: PCORI Engagement Rubric. PCORI (Patient-Centered Outcomes Research Institute) website. http://www.pcori.org/sites/default/files/Engagement-Rubric.pdf. Published February 4, 2014

This unique educational portfolio developed by the core engagement team is classified into two categories: education about community engagement strategies to support engagement efforts; and specific training at key research phases within PCOR (Fig. 1). All educational modules were offered via the Zoom platform, which increased reach and was particularly valuable during the COVID-19 pandemic in overcoming participation barriers. Interactive breakouts and the use of features such as chat, annotation, whiteboard, and relevant vignettes/case studies were utilized to increase participation, bolster discussion, and strengthen knowledge transfer.

### AHeaD with CHWs in COVID-19

Although CHWs are uniquely poised to help address health problems created or exacerbated by public health crises, as well as engage minorities in research related to these resultant health disparities [14], they have been largely underutilized to understand and help address COVID-19 disparities from a PCOR perspective [15]. Therefore, CHWs participated in time-sensitive training to equip them with skills to overcome challenges when engaging with the community during the COVID-19 pandemic. Specific educational curricular focused on: (1) motivational interviewing during COVID-19 to overcome barriers to change, with specific emphasis on how to ask open-ended questions, provide affirmations, demonstrate reflective listening, and effectively summarize community concerns; (2) myth-busting to learn about best practices to debunk misconceptions circulating in the community and hindering health while respecting one’s beliefs; and (3) creative community engagement strategies to addresses face-to-face barriers resulting from the COVID-19 pandemic (i.e., utilization of cloud-based telecommunication platforms, graphic medicine, gamification, social media, etc.). Curriculum was developed by drawing from SAB and CHW Advisory Council expertise as well as resources from the National Association of CHWs and the World Health Organization. Additionally, sessions were co-facilitated by an experienced CHW who is the lead instructor for a local CHW training institute and has a deep understanding of the needs and interests of the attendees. Since adults learn best when content is relevant, practical, and they can draw from their own experiences actively throughout the learning process [5,6], the curriculum was organized in a way that allowed for continuous feedback, knowledge checks, and time points for sharing and troubleshooting challenges experienced. This problem-based learning approach [18] was particularly helpful as CHWs shared barriers to reaching their community during pandemic-enforced lockdowns. Additional training topics were offered to help advance CHWs knowledge of PCOR concepts and key research processes, including informed consent, study randomization, and results dissemination.

### PaTH to Heath: Diabetes

Engagement activities in the PaTH to Health: Diabetes study has been described elsewhere [16,17]. This section will focus specifically on the educational opportunities offered to stakeholders on this project. To cultivate a safe space for learning and honor the significance of stakeholder contributions, the PaTH to Health: Diabetes study team dedicated the study kick-off meeting to underscore PCOR engagement principles (reciprocal relationships, co-learning, partnerships, transparency, honesty, and trust) as the foundation for successful partnership, and these were reinforced during subsequent meetings and educational programming [16,19]. These principles ensure a level playing field regardless of educational or professional background so all partners can feel empowered to provide feedback; support bidirectional learning; demonstrate value of partnerships through appropriate reimbursement for contributions; and maintain inclusive decision-making.

In addition, partners attended Just-In-Time (JIT) trainings to address foundational knowledge gaps as the project entered key research phases, including research ethics, data extraction, data analysis, and results dissemination. Content focused on critical considerations in observational research, including how electronic health record (EHR) data can be used to answer research questions, the importance of data standardization and data security, and how study results can be shared with scientific and lay audiences. These interactive modules developed by project staff with senior research experience and adult education training included lay language, clear objectives, and opportunities for case analysis, group brainstorming, and debriefing [6]. Trainings were offered at the beginning of the respective research phase so stakeholders could rapidly draw from content and feel empowered to engage in future research meetings regarding the topic.

## Methods of Evaluation

The AHeaD with CHWs in COVID-19 project team adapted Kirkpatrick’s Four Levels of Training Evaluation (reaction, learning, impact, results) [20] to evaluate the CHW virtual community engagement training. Survey evaluations were administered at three time points (pre-training, immediately post-training, and 1 month post-training) to assess learner’s perception of training quality; skills, confidence, and knowledge regarding PCOR processes; and preparation to engage and apply PCOR skills remotely. The 1 month post-training evaluation included a 5-point Likert scale to assess how the training affected participants’ behaviors and long-term outcomes. Pre/post-knowledge of PCOR concepts and research topics offered in the PCOR training were also assessed.

In the PaTH to Health: Diabetes study, stakeholders had the opportunity to participate in yearly quantitative and qualitative evaluations assessing the breadth of engagement activities, including education offerings, from the prior project period. This data helped elucidate the effectiveness of the research team’s involvement of stakeholders and of training and other engagement strategies as enhancements to stakeholder contributions to the project.

All quantitative evaluation data were collected and managed using Research Electronic Data Capture (REDCap) hosted at the Penn State University College of Medicine.

## Initial Evidence of Impact on Learning

Roughly half of the training attendees in the AHeaD with CHWs in COVID-19 project completed the pre-training evaluation, though this was not mandatory (n = 51), and 29 participants completed the immediate post-training evaluation. We found evidence of impact on learning through increased community engagement reported by training participants. When comparing pre- and immediate post-training survey results (n = 15) using paired sample *t*-tests, there were statistically significant increases in reported knowledge of and confidence in virtual community engagement strategies, COVID-19 resources, motivational interviewing methods, and myth-busting techniques (Table 2). Participants who completed the 1 month post-training evaluation (*n* = 13) reported that they used the motivational interviewing techniques and community engagement strategies in their work (mean 4.54 ± 0.88; mean 4.62 ± 0.51, respectively). They also reported increased confidence in utilizing myth-busting strategies (mean 4.85 ± 0.38), an important skill given the multitude of misinformation creating barriers to health during a pandemic. Further, CHWs improved in their knowledge of PCOR concepts from a low–moderate pre to a moderate–high post-knowledge level.

**Table 2.** AHeaD with CHWs^a^ in COVID-19 pre- and immediate post-change in mean confidence and knowledge level

Variable	N	Mean	Mean difference	95% CI
Confidence in virtual strategies	15	Pre: 2.73; Post: 4.13	1.40^b^	[0.65, 2.15]
Knowledge of COVID-19 resources	15	Pre: 3.06; Post: 4.00	0.93^b^	[0.29, 1.58]
Knowledge of motivating interviewing techniques	15	Pre: 2.67; Post: 4.13	1.47^b^	[0.96, 1.97]
Knowledge of myth-busting techniques	15	Pre: 2.27; Post: 4.13	1.86^b^	[1.27, 2.45]

a Community Health Workers.

b P < 0.01.

The PaTH to Health: Diabetes study also found evidence of impact on learning through enhanced engagement in research activities. For example, after partners attended a data extraction training to better understand how EHR data is used for research purposes while maintaining patient confidentiality, partners co-developed an interactive whiteboard video and dissemination brief on this topic. These patient-facing resources were helpful in mitigating the stigma associated with research by describing how health information can be used safely to answer important research questions. Similarly, after attending results dissemination training on the myriad ways to communicate research results to both scientific and lay audiences, patient partners assisted in the development of lay public dissemination briefs of published study manuscripts. Partners were helpful in pulling out key manuscript highlights and conveying these sections in lay-appropriate language, with visuals to display study findings. These one-pagers effectively translate scientific findings to a general audience, underscoring the important role partners can play in disseminating results to the broader community (see Appendix). Additionally, several stakeholders participated in scientific communications, including presenting updates at network meetings and serving as co-authors on study publications.

## Conclusions

Standard research training programs are lackluster in addressing the unique needs of stakeholders partnering in PCOR efforts. This report highlights the varying approaches to stakeholder education that may be appropriate depending on the type of stakeholder and level of engagement in research, underscoring it is not a one-size-fits-all approach. Given our limited evaluation data, further research is needed to understand how educational efforts continue to impact stakeholders post-training and if a more equipped stakeholder workforce facilitates enhanced community–researcher partnerships.

## Supplementary material

For supplementary material accompanying this paper visit http://dx.doi.org/10.1017/cts.2021.835. click here to view supplementary material
