# Supplementary material for: Enhancing community engagement in Patient-Centered Outcomes Research: Equipping learners to thrive in translational efforts
Source: J Clin Transl Sci. 2021 Aug 13;5(1):e172. doi: 10.1017/cts.2021.835 (PMC8532187; doi:10.1017/cts.2021.835)
Supplement: Supplementary file 1 [file S2059866121008359sup001.pdf]

PaTH TO HEALTH *diabetes*

PennState College of Medicine

Appendix: Lay Dissemination Briefs

Penn State College of Medicine
PATHtoHealth@pennstatehealth.psu.edu
www.pathdiabetes.org

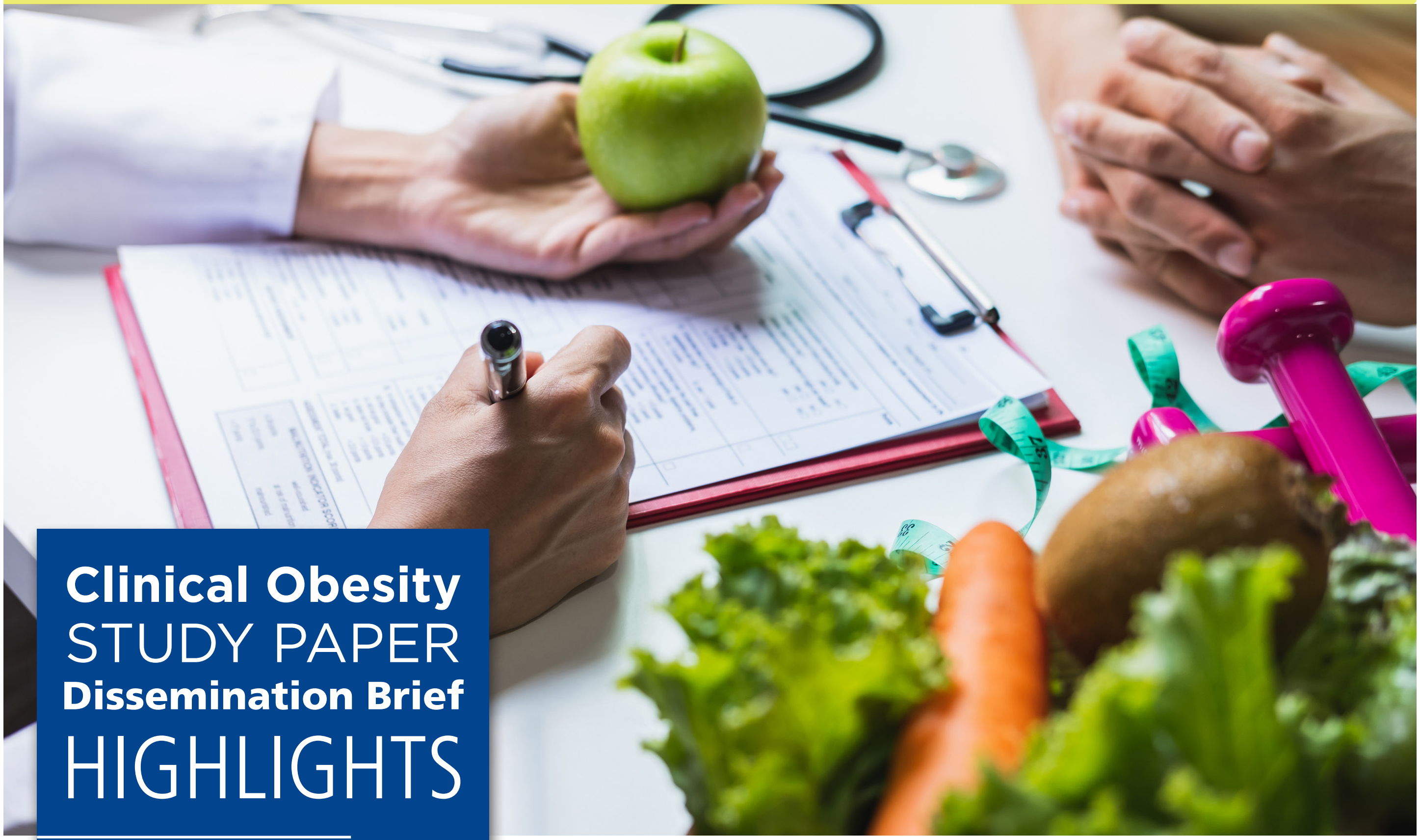

Clinical Obesity STUDY PAPER Dissemination Brief HIGHLIGHTS

Utilization of Intensive Behavioral Treatment for Obesity in Patients with Diabetes

OBESITY = A LEADING PUBLIC HEALTH CONCERN

Overweight and obesity are considerable issues in the U.S. Addressing obesity through lifestyle interventions (i.e. healthy eating and increased physical activity) decreases the risk of developing type 2 diabetes, a disease that affects nearly 29 million people (9.3% of the U.S. population). Diabetes is associated with serious complications, including heart disease, blindness, renal failure and lower extremity amputation. Although complications are preventable with proper medical and lifestyle management, including weight loss, nearly half of patients with diabetes do not have adequate glycemic control.

WHAT'S BEING DONE **ABOUT OBESITY?**

Recent policy changes and health insurance expansion have resulted in universal coverage for obesity counseling to improve weight loss for adults either with or at high risk of type 2 diabetes. Eligible patients can receive up to 20 face-to-face visits for obesity counseling in the primary care setting. However, whether or not this new benefit has actually helped patients better control their diabetes and lose weight is unknown.

THAT'S WHERE **PATH TO HEALTH: DIABETES STUDY** COMES IN!

The goal for the PaTH to Health: Diabetes Study is to understand how often obesity counseling is being provided to patients with a diabetes diagnosis from 2009-2017.

Flip to learn more about the study.

PennState Health

TEMPLE HEALTH

JOHNS HOPKINS MEDICINE

University of Pittsburgh

Geisinger

UNIVERSITY OF UTAH HEALTH SCIENCES

WHERE IS THE **DATA** FROM?

Study members access electronic health record data from the PaTH Clinical Data Research Network (CDRN), a partnership of six health systems across three states that include the following health organizations: Penn State Health, University of Pittsburgh, Temple University, Geisinger Health System, Johns Hopkins University and University of Utah. Through this network, study personnel are able to access de-identified health data – which means a person's health information cannot be linked to their identity. For the clinical obesity paper, the study reported how often obesity counseling was used on a yearly basis for patients. Descriptive information was captured about patients who received counseling, including their gender, race, age and if they lived in urban vs. rural areas.

WHAT DID THE STUDY **FIND**?

A total of **219,276 patients** were included in the study analyses. Findings showed that a minimal number of patients are receiving obesity counseling – only 0.27%. Data displayed that patients who received obesity counseling tended to be:

FEMALE

NON-WHITE
(black or Hispanic)

YOUNGER
less than 65 years of age

reside in **URBAN AREAS**

To learn more about how health data is collected and protected, visit **http://bit.ly/De-identifyingData**

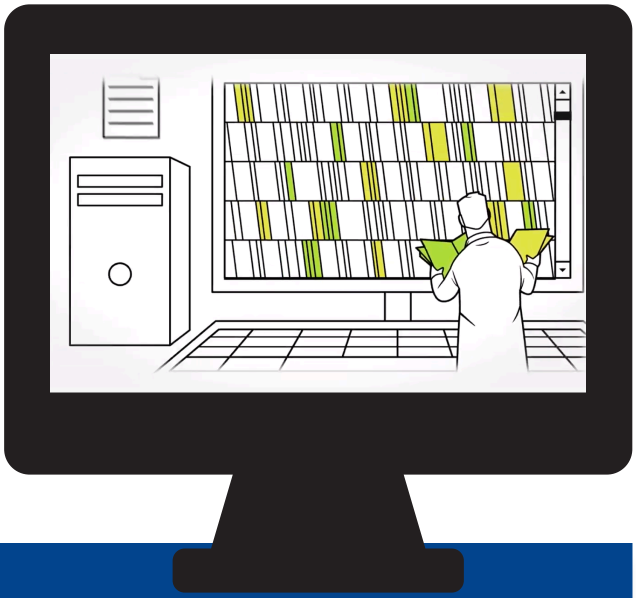

WHAT IS THE NEXT STEP?

Overall, obesity counseling for patients with diabetes remains low. Future work is necessary to understand the impact of this counseling and, if effective, how to increase use within primary care.

Kraschnewski J, Yeh H, Francis E, Kong L, Lehman E, Rovito S, Poger J, Bryce C. 2019 Utilization of Intensive Behavioral Treatment for Obesity in Patients with Diabetes. Clin Obes. 2021;11:e12426. doi/10.1111/cob.12426.

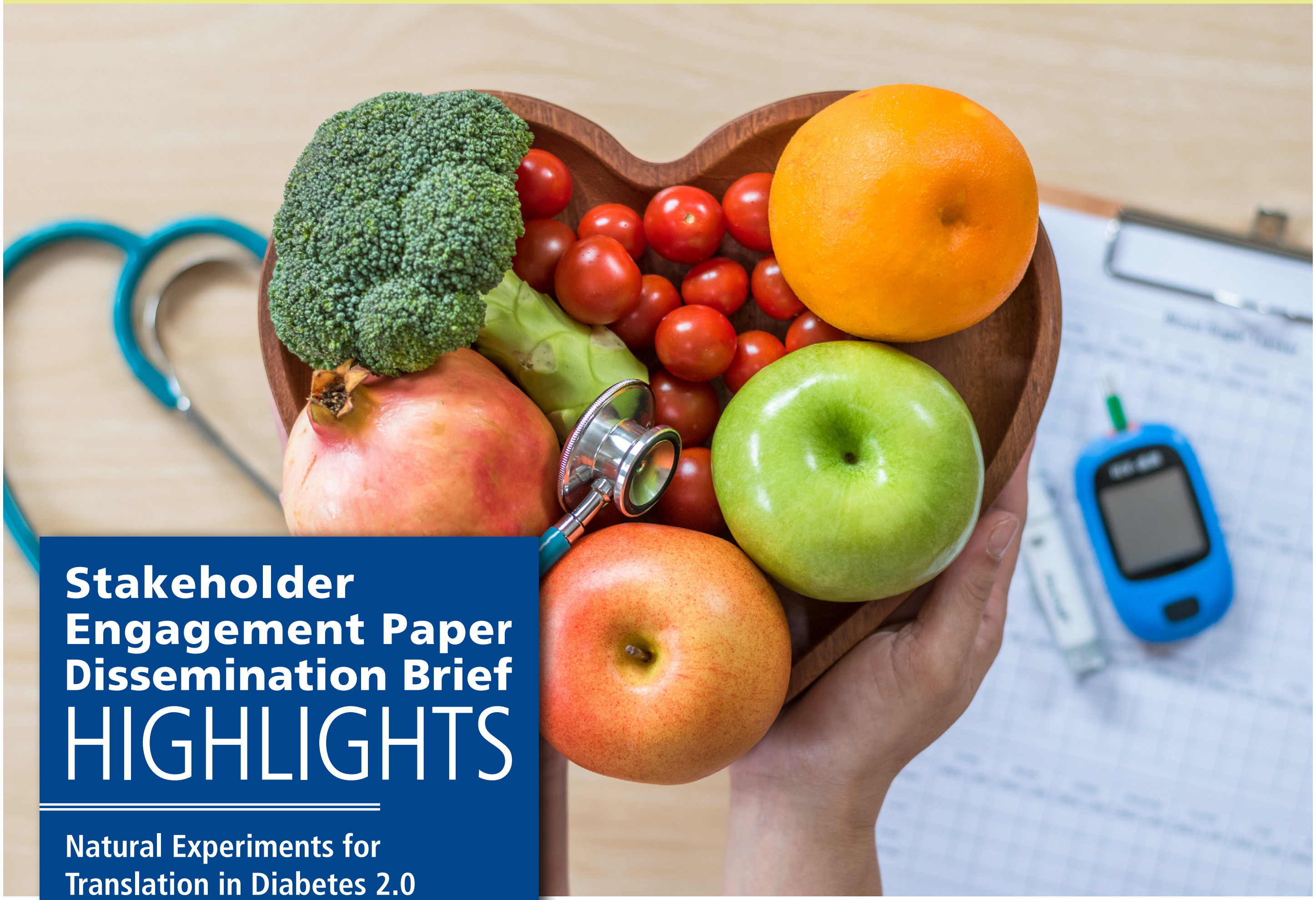

Stakeholder Engagement Paper Dissemination Brief HIGHLIGHTS

Natural Experiments for Translation in Diabetes 2.0 (NEXT-D2) Network Engagement In Action: Stakeholder Engagement Activities to Enhance Patient-Centeredness of Research

SHIFT IN HOW WE DO RESEARCH

Instead of conducting research *on* the community, research teams are increasingly conducting research *with* the community. There are many valuable contributions community partners can make to enhance a research study. Research teams can work with community partners to select research questions that patients care most about, include their feedback in study decisions, and share study results with relevant populations to improve health.

WHO ARE **COMMUNITY PARTNERS?**

Community partners (also referred to as stakeholders) can include anyone who has a voice to share in the research: patients, caregivers, policy makers, clinicians, advocacy groups, health system leaders, health insurance providers, and others.

WHY DOES THIS **MATTER?**

The ultimate goal of research on individuals is to improve lives. Inviting the community to engage in the research table and engaging them throughout the research process can help ensure we are asking the right research question(s), improve how we run the study, and keep patients at the center of our work.

Flip to learn more about the study.

WHAT ARE **NATURAL EXPERIMENTS?**

In a natural experiment, researchers do not change patients' care – instead, they closely watch what is happening in the real world to find out if treatment plans and programs that currently exist are actually helping people. This is different from other studies, such as clinical trials, where individuals are placed in either a treatment or placebo group by the researchers.

Natural experiments allow us to study health information on a LOT of people – much more than a traditional research study. This means we can better understand if what may be helping a large group of people would also be the case for the greater good.

HOW CAN COMMUNITY PARTNERS **HELP WITH NATURAL EXPERIMENTS?**

Community partners can provide feedback to research teams that can positively impact a research study, including how a study is set up, how it is conducted, what outcomes matter most to the community, and how to share the resulting information with the community.

Natural experiments in particular can benefit from community input by addressing real world issues that affect patients and providing insight into future policy decisions.

HOW DID WE DO IT?

Our network, the Natural Experiments for Translation in Diabetes 2.0 (NEXT-D2), has been established to help identify programs and policies that are most helpful to patients with diabetes and prediabetes. NEXT-D2 consists of **8 partnering institutions** who are each conducting a natural experiment related to diabetes and who engage the community in their study. NEXT-D2 formed an Engagement committee for partnering institutions to meet regularly to **share engagement resources and approaches** that have been successful with their study's community partners. Key community engagement activities at the partnering institutions include Study Meetings, Proposal Development, Trainings & Educational Opportunities, Data Analysis, and Results Dissemination.

Network-wide **patient-centered resources and multimedia** have also been developed by engaging each institutions' community groups. This collaboration has been beneficial in supporting **meaningful stakeholder engagement** in natural experiments.

ENGAGEMENT COMMITTEE:
Stakeholder Participation

STUDY MEETINGS
updates, brainstorming, shared decision-making, and planning

PROPOSAL DEVELOPMENT
study design and goals

EDUCATIONAL OPPORTUNITIES
research process trainings

DATA ANALYSIS
interpretation and evaluation of research findings

RESULTS DISSEMINATION
presenting research results, assisting in paper-writing, developing community resources

Poger JM, Mayer V, Duru OK, Nauman B, Holderness H, Warren N, Vasquez C, Bibi S, Rasmussen-Torvik LJ, Hosseinian Z, Shi L, Wallace J, Goytia CN, Horowitz CR, Kraschnewski JL. Natural Experiments for Translation in Diabetes 2.0 (NEXT-D2) Network Engagement In Action: Stakeholder Engagement Activities to Enhance Patient-Centeredness of Research. Med Care. 2020;58 Suppl 6 Suppl 1:S66-S74. doi:10.1097/MLR.0000000000001264.
